# Catecholaminergic Regulation of Learning Rate in a Dynamic Environment

**DOI:** 10.1371/journal.pcbi.1005171

**Published:** 2016-10-28

**Authors:** Marieke Jepma, Peter R. Murphy, Matthew R. Nassar, Mauricio Rangel-Gomez, Martijn Meeter, Sander Nieuwenhuis

**Affiliations:** 1 Cognitive Psychology Unit, Institute of Psychology, Leiden University; and Leiden Institute for Brain and Cognition, Leiden University, Leiden, the Netherlands; 2 Department of Neurophysiology and Pathophysiology, University Medical Center Hamburg-Eppendorf, Hamburg, Germany; 3 Department of Cognitive, Linguistic & Psychological Sciences, Brown University, Providence, RI, United States of America; 4 Department of Psychology, University of California, Berkeley, United States of America; 5 Department of Education Sciences, Vrije Universiteit Amsterdam, The Netherlands; Oxford University, UNITED KINGDOM

## Abstract

Adaptive behavior in a changing world requires flexibly adapting one’s rate of learning to the rate of environmental change. Recent studies have examined the computational mechanisms by which various environmental factors determine the impact of new outcomes on existing beliefs (i.e., the ‘learning rate’). However, the brain mechanisms, and in particular the neuromodulators, involved in this process are still largely unknown. The brain-wide neurophysiological effects of the catecholamines norepinephrine and dopamine on stimulus-evoked cortical responses suggest that the catecholamine systems are well positioned to regulate learning about environmental change, but more direct evidence for a role of this system is scant. Here, we report evidence from a study employing pharmacology, scalp electrophysiology and computational modeling (N = 32) that suggests an important role for catecholamines in learning rate regulation. We found that the P3 component of the EEG—an electrophysiological index of outcome-evoked phasic catecholamine release in the cortex—predicted learning rate, and formally mediated the effect of prediction-error magnitude on learning rate. P3 amplitude also mediated the effects of two computational variables—capturing the unexpectedness of an outcome and the uncertainty of a preexisting belief—on learning rate. Furthermore, a pharmacological manipulation of catecholamine activity affected learning rate following unanticipated task changes, in a way that depended on participants’ baseline learning rate. Our findings provide converging evidence for a causal role of the human catecholamine systems in learning-rate regulation as a function of environmental change.

## Author Summary

Belief updating in response to changes in the environment is crucial for adaptive behavior. We examined the role of the human catecholamine (norepinephrine and dopamine) systems in this process, using a combination of pharmacology, scalp electrophysiology and computational modeling. We found that the P3 component of the event-related potential—an electrophysiological index of phasic catecholamine release in the cortex—predicted learning rate and mediated the effect of prediction-error magnitude on learning rate. Furthermore, a pharmacological manipulation of catecholamine activity affected learning rate following unanticipated task changes, in a way that depended on participants’ natural learning rate. These findings may reflect the catecholaminergic regulation of belief updating following environmental change.

## Introduction

The ability to adapt to a changing world is fundamental for survival, and requires the updating of beliefs in response to unexpected events that signal potential environmental change [1]. In reinforcement-learning models, belief updating is driven by prediction errors, which are formalized as the difference between predicted and actual outcomes. Moreover, the degree to which each prediction error influences existing beliefs depends on the learning rate, such that beliefs are updated according to the following ‘delta rule’: Belief_(t+1)_ = Belief_(t)_ + learning rate× prediction error_(t)_ [2]. In this way, learning rate determines the relative influence of recent compared to more historical events on current beliefs. Thus, noisy but otherwise static environments require low learning rates, resulting in stable beliefs, whereas rapidly changing (i.e., volatile) environments require higher learning rates and more flexible beliefs [3,4].

Neuroimaging studies have identified brain areas associated with belief updating and with various factors that drive learning rate, including the volatility and uncertainty associated with the task environment and the surprise elicited by an outcome [3,5–8]. However, a critical question that remains to be addressed concerns the role of neuromodulatory systems in the regulation of learning rate. Several lines of evidence have pointed towards a role of norepinephrine (NE) and dopamine (DA) in belief updating. At a cellular level, NE increases the responsivity of its target neurons to their afferent input [9]. Furthermore, the effects of NE on synaptic transmission within cortical structures are thought to favor the processing of external sensory stimuli over intrinsic top-down information [10], which makes NE well-positioned to regulate learning rate based on environmental change. Indeed, theoretical and modeling work has suggested that NE signals *unexpected uncertainty* arising from unsignaled changes in a task context, whereas it is not sensitive to the *expected* unreliability of outcomes [11]. A lesion study in rats has provided the first evidence for a role of the noradrenergic system in basic reward learning [12], and findings that pharmacological manipulations and lesions of the noradrenergic system affect reversal learning and attentional-set shifting performance in animals [13–19] are broadly consistent with the idea that the noradrenergic system is involved in detecting environmental change. However, these studies do not provide specific evidence for a role of this system in learning-rate regulation. The most specific empirical evidence to date for a role of the human noradrenergic system in learning-rate regulation comes from studies that found correlations between learning rate [20–22] or prediction error [23], and pupil size, an indirect index of locus coeruleus (LC; the main noradrenergic nucleus of the brain) activity [24–28].

DA has also been implicated in the regulation of learning rate [29]. Like NE, DA has the potential to modulate brain-wide synaptic transmission. The view that DA plays an important role in learning-rate regulation is also consistent with studies indicating that DA neurons respond to novel and unexpected stimuli, and that DA is critical for cognitive flexibility [30,31]. More specific evidence is provided by two studies that have linked individual differences in learning rate to the gene polymorphisms coding for COMT, the DA transporter, and D2 receptors [32,33]. Although the COMT enzyme metabolizes both DA and NE, its effect on NE levels is thought to be minor compared to that on DA, at least in the prefrontal cortex [34].

In the present research we examined evidence for a role of the human catecholamine systems in learning-rate regulation, using a well-established ‘predictive-inference’ task that provides direct measures of prediction error and learning rate on each trial [4,21]. First, we used the centroparietal P3 component of the EEG as an electrophysiological correlate of outcome-evoked phasic catecholamine release in the cortex [35–38] (but see the Discussion for a competing hypothesis), and examined trial-by-trial relationships between prediction-error magnitude, P3 amplitude and learning rate using multilevel mediation analyses. Second, we examined the relationships between P3 amplitude and two latent variables—capturing the unexpectedness of an outcome and the uncertainty about the outcome-generating process—that together determine learning rate according to a previously established normative model [4,5,21]. Third, we examined the causal effects of a pharmacological manipulation of catecholamine activity on learning rate. Note that we used the correlational P3 analyses and the pharmacological manipulation as two independent approaches, which together could provide converging evidence regarding the role of the catecholamine systems in learning-rate regulation.

## Results

**Fig 1:**
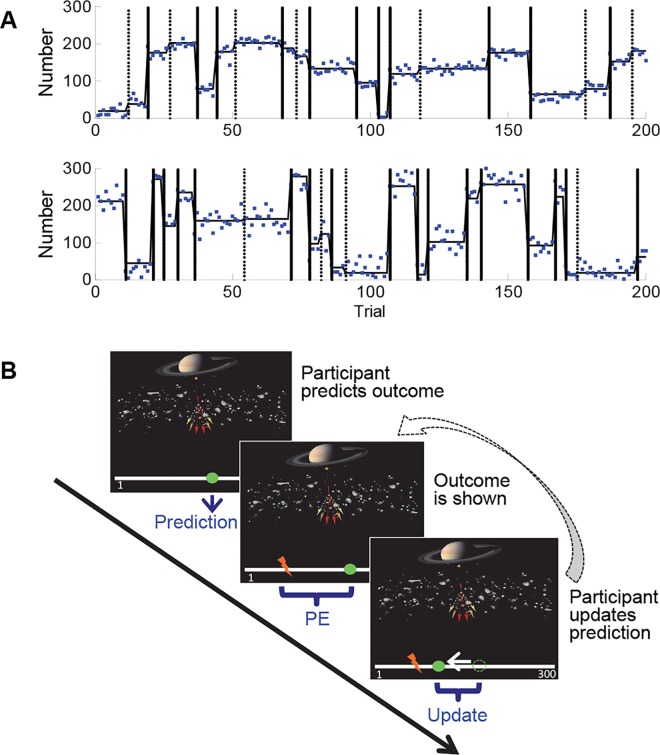
The predictive-inference task. A. The number outcomes in a low-noise (upper panel) and a high-noise (lower panel) block. The blue dots indicate the number outcomes on each trial; these were drawn from a normal distribution of which the mean (horizontal black line) changed at unsignaled moments (change points). Change points are indicated by the vertical lines, separately for obvious (straight lines) and non-obvious (dotted lines) change points. The SD of the number-generating distribution was constant within each block, but varied between blocks (either 10 or 25). B. Illustration of the task. On each trial, participants predict the next location on a number line, after which they see the actual location (outcome) and update their prediction for the next trial. The number-line locations correspond to the number outcomes shown in plot A.

Participants performed a predictive-inference task in which they repeatedly predicted the next location on a number line [4,21]. The location (i.e., number) on each trial was drawn from a Gaussian distribution. The standard deviation of this distribution was 10 (low noise) or 25 (high noise), in separate blocks, and the mean of the distribution changed at unpredictable intervals—referred to as change points (Fig 1A; see Methods for more details of the number-generating process). We embedded the task in a cover story according to which the number line represented the earth, the number outcomes reflected the locations of missile attacks from outer space, and on each trial participants could place a “laser shield” above a specific location on earth to prevent that location from being hit (Fig 1B). This task allows direct observation of trial-specific prediction errors (the difference between the predicted and observed outcome) and learning rates (the prediction update from one trial to the next, as a fraction of the most recent prediction error; cf. [4]). We recorded participants’ EEG during the task, and determined the amplitude of the outcome-evoked P3 on each trial. Participants completed the task twice, on separate days. On one day, participants received a placebo pill. On the other day, they received a single dose of 40 mg atomoxetine, a selective NE transporter (NET) blocker. Within the cortex NET is also responsible for DA reuptake, due to the paucity of DA transporters in the cortex [39]. Thus, NET blockers increase both central NE and cortical DA availability [40–43].

Below, we will report three sets of analyses and results. First, we will report the within-subject relationships between trial-to-trial fluctuations in prediction error, P3 amplitude, and learning rate. Second, we applied a normative learning model that has been shown to capture key aspects of participants’ performance on this task [4,5,21] to estimate the latent variables that drive learning rate in this task. The normative model uses the sequence of observed outcomes to compute two latent variables (change-point probability and relative uncertainty) on each trial. Together, these latent variables in turn determine trial-specific learning rate (see Methods for more details on the model). We will report the within-subject relationships between these latent variables, P3 amplitude, and learning rate. Third, we will report the effects of our atomoxetine manipulation.

Note that whereas we used P3 amplitude as a correlate of phasic catecholamine release, the exact effects of atomoxetine on phasic vs. tonic catecholamine activity are unknown. Although microdialysis studies have shown that a single dose of atomoxetine or reboxetine (another NET blocker) increases catecholamine concentrations in the rat brain [44,45], it is unknown whether this reflected an increase in tonic and/or phasic activity, due to the limited temporal resolution of microanalysis. Thus, atomoxetine may affect tonic and/or phasic catecholamine activity, and which of these two effects dominates is unknown. To foreshadow the atomoxetine results, there were no atomoxetine effects on learning rate or P3 amplitude at the group level, but analyses of individual differences revealed that atomoxetine affected learning rate in a baseline-dependent manner.

### P3 amplitude predicts learning rate and mediates the effect of prediction error on learning rate

**Fig 2:**
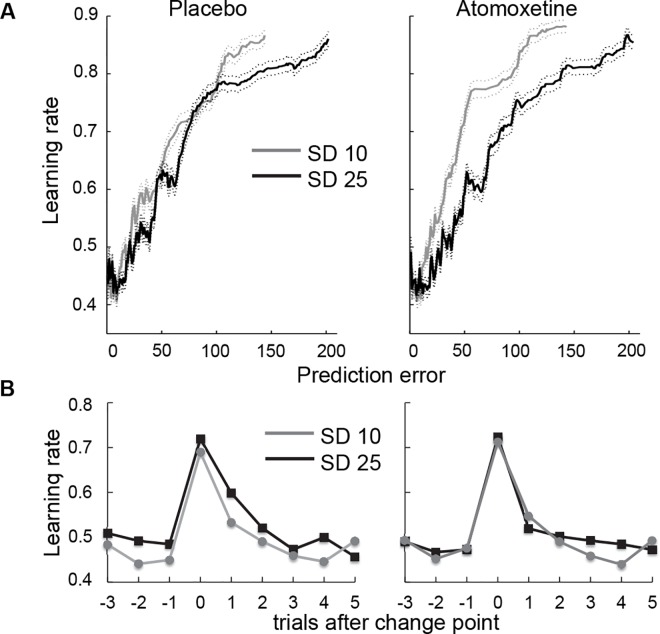
Learning rate increases as a function of prediction error and following change points. A. Learning rate as a function of prediction error, SD of the generative distribution and treatment. Learning rate is plotted as a function of median absolute prediction error, averaged using running bins of 150 trials, pooled across participants. Solid and dashed lines indicate mean and SEM, respectively. Running bins with the same median prediction error were combined, and the average learning rate was computed across the resulting larger bin. Note that although we used running-average bins for plotting purposes, we used single-trial measures of prediction error and learning rate in our statistical analyses (see Methods). B. Learning rate as a function of trials after change point, SD of the generative distribution and treatment (left = placebo, right = atomoxetine).

Consistent with previous studies [4,21], learning rate increased with increasing prediction error (*t* = 9.6, *p* < .001), and this effect was stronger in the low-noise than the high-noise block (prediction error x noise interaction, *t* = 5.9, *p* < .001; Fig 2A). These effects are consistent with optimal task performance as prediction error magnitude is an important predictor of whether or not a change point occurred—especially when the SD of the generative distribution is low—and hence whether or not an increase in learning rate is warranted to discount the influence of previous outcomes on beliefs. Indeed, learning rate increased immediately following a change point and gradually decayed during the subsequent trials when participants adjusted their predictions to the new outcome distribution (Fig 2B). Note that the optimal relationship between prediction error magnitude and learning rate depends on the stability and noise level of the environment: an increase in learning rate with increasing prediction error is advantageous in environments with occasional abrupt changes—as was the case in our task—but not in noisy but otherwise stable environments.

**Fig 3:**
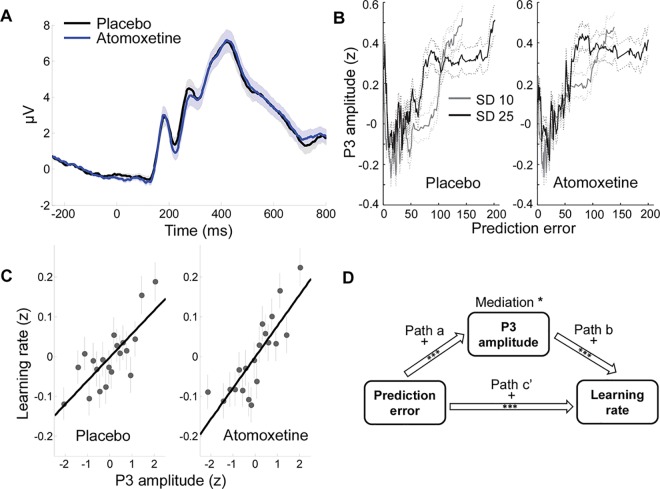
Relationships between prediction error, P3 amplitude and learning rate. A. Grand-average outcome-evoked P3 (average signal across a cluster of four centroparietal electrodes centered on locations CPz and Pz), in the placebo and atomoxetine session. B. P3 amplitude as a function of prediction error, SD of the generative distribution and treatment. To make these plots, single-trial P3 amplitudes were *z*-scored per participant and plotted as a function of median absolute prediction error, averaged using running bins of 150 trials, pooled across participants. Running bins with the same median prediction error were combined, and the average P3 amplitude was computed across the resulting larger bin. Solid and dashed lines indicate mean and SEM, respectively. C. P3 amplitude predicts learning rate. *Z*-scored learning rates are sorted into 20 bins according to *z*-scored single-trial P3 amplitude, pooled across participants. The lines show the linear fit to the unbinned single-trial data. D. Mediation model and results. *** *p* < .001, * *p* < .05.

The number outcomes evoked a strong P3, of which the amplitude was maximal at centroparietal electrodes (Fig 3A). Like learning rate, the amplitude of the outcome-evoked P3 increased with increasing prediction error (*t* = 4.2, *p* < .001; Fig 3B), but there was no significant main effect of noise (*p* = .09) and no prediction error x noise interaction on P3 amplitude (*p* = .12). The positive effect of prediction error on P3 amplitude corroborates a large body of evidence that the P3 is highly sensitive to the subjective probability [46] or surprise [47] of the eliciting stimulus. In addition, consistent with previous pupil dilation findings on this task [21], P3s were relatively large on trials when prediction error was exactly 0 (*t* = 4.2, *p* < .001; Fig 3B), possibly reflecting the rewarding nature and/or atypical consequence (i.e., no possibility of updating the next prediction) of perfectly predicted outcomes. Finally, Fig 3B suggests that P3 amplitude was highly sensitive to variation in prediction-error magnitude within the range of low prediction errors, but less so within the range of very high prediction errors. This may be related to the fact that all prediction errors above ~80 in our task were associated with change-point probabilities close to 1.

We next used multi-level mediation, a recently developed path analysis method (e.g., [48,49]), to test whether P3 amplitude is predictive of learning rate when controlling for prediction error, and whether P3 amplitude mediates the relationship between prediction error and learning rate (see Methods for more details on mediation analyses).

The effect of prediction error on P3 amplitude was as described above (path *a*, *p* < .001). Importantly, larger P3s predicted higher learning rates when controlling for prediction error (path *b*, *p* < .001; see Fig 3B for the relationship between P3 amplitude and learning rate, not controlled for prediction error). In addition, P3 amplitude formally mediated the effect of prediction error on learning rate (a*b, *p* = .02; Fig 3C). When controlled for P3 amplitude, the relationship between prediction error and learning rate remained highly significant (path *c’*, *p* < .001), implying a partial rather than a full mediation.

Together, these results suggest that (i) the amplitude of an outcome-evoked P3 predicts the degree to which that outcome influences existing beliefs; and (ii) prediction errors influence learning rate in part via a process that is reflected in P3 amplitude. As the P3 is a correlate of phasic catecholamine release in the cortex, these findings provide support for a role of the catecholamine systems in incorporating new unexpected observations into beliefs about the causal structure of the environment. Note that the absence of a full mediation suggests that there is an additional effect of prediction error on learning rate that is not mediated by P3 amplitude (see Discussion).

Finally, to examine the specificity of these effects to the centroparietal P3, we conducted control mediation analyses testing for possible ERP mediators of the prediction error-learning rate effect across the entire space of scalp locations and time points. Specifically, in separate analyses, we used the mean EEG signal at each electrode, during each 20-ms time interval following outcome onset (spanning the 0–600 post-outcome period) as mediators. These analyses revealed only two ERP components that were significant mediators of the prediction error-learning rate relationship: (i) a 360–560 ms post-outcome centroparietal signal, corresponding to the P3 examined in detail above; and (ii) a 160–200 ms post-outcome centro-occipital signal, corresponding to the P2. In contrast to the P3, the P2 component was negatively related to both prediction error and learning rate (the negative relationship with prediction error was apparent as early as ~100 ms post-outcome). The negative effect of prediction error on early occipital potentials likely reflects spatial-attention effects related to the spatial character of our task: prediction-error magnitude was perfectly correlated with the distance between the predicted and actual outcome location on a number line. Thus, outcomes that produced larger prediction errors appeared at spatial locations that were less likely to be attended at the time of outcome presentation. Given the large body of evidence that stimuli at attended vs. unattended locations elicit larger early visual-evoked potentials over occipital electrodes [50], the above implication of the P2 may reflect spatial attention effects driven by outcomes arriving further away from participants’ predicted locations.

### P3 amplitude reflects both change-point probability and relative uncertainty

So far, we focused on the effect of directly observable prediction errors on learning rate, and its mediation by P3 amplitude. Next, we examined two latent variables that drive trial-to-trial fluctuations in learning rate according to a previously established normative model [4,5,21]. Rather than assuming a direct effect of prediction error on learning rate, the model assumes that participants use the observed sequence of outcomes to compute two latent variables on each trial, change-point probability and relative uncertainty, which together determine learning rate (see Methods section for model details). Change-point probability approximates the posterior probability that a change point has occurred since the previous trial, given all previous observations; hence it reflects the unexpectedness of an observation. As expected, the trial-to-trial changes in change-point probability and prediction error were strongly correlated in all participants (correlation ranges from .75 to .85, all *p*’s < .001). Relative uncertainty reflects the uncertainty about the mean of the outcome distribution *before* a new outcome is observed, which depends inversely on the number of prior observations attributable to the current environmental state. Thus, one important difference between relative uncertainty and change-point probability is that they are computed pre- and post-outcome respectively. Relative uncertainty did not correlate with prediction error in most participants (correlation ranges from -.01 to .15; *p* > .05 for 20 of the 27 participants). Moreover, the correlation between change-point probability and relative uncertainty was negative but non-significant for most participants (correlation ranges from -.07 to -.04; *p* > .05 for 24 of the 27 participants). The determination of learning rate by change-point probability and relative uncertainty captures the idea that belief updating should be stronger following outcomes that are likely to signal environmental change, and when the current state of the environment is uncertain, respectively.

**Fig 4:**
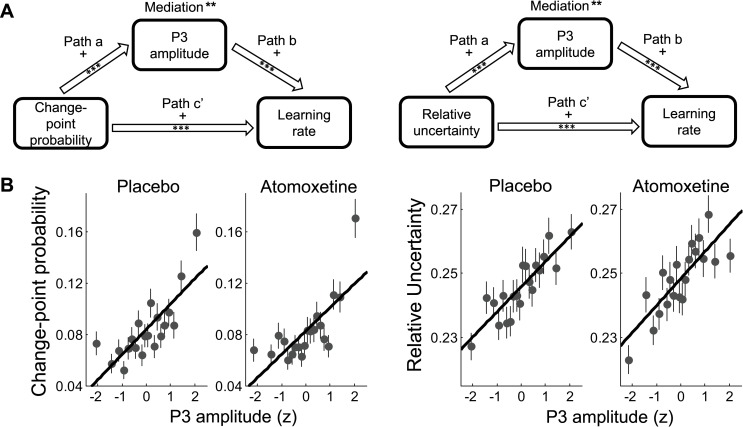
Relationships between change-point probability and relative uncertainty, P3 amplitude and learning rate. A. Mediation models and results. *** p < .001, ** p < .01. B. Both change-point probability and relative uncertainty predict P3 amplitude. Change-point probability and relative uncertainty estimates are sorted into 20 bins according to z-scored single-trial P3 amplitude, pooled across participants. The lines show the linear fit to the unbinned single-trial data.

We examined the relationship between trial-by-trial fluctuations in each of these two latent variables and P3 amplitude in two additional mediation analyses. In these analyses we used either change-point probability or relative uncertainty, rather than prediction error, as the independent variable (Fig 4A). Interestingly, both change-point probability and relative uncertainty were positive predictors of P3 amplitude (path *a*, both *p*’s < .001; Fig 4B). Given the strong relationship between change-point probability and prediction error, the positive effect of change-point probability on P3 amplitude was to be expected. That relative uncertainty was also predictive of P3 amplitude suggests that P3 amplitude reflects not only the unexpectedness of an outcome, but also the pre-existing uncertainty about the mean of the outcome distribution and hence the informational value of the outcome for improving prediction accuracy. Moreover, P3 amplitude formally mediated the effect of both change-point probability and relative uncertainty on learning rate (path a*b, *p* = .003 and .001, respectively).

### Atomoxetine effects on learning rate following change points depend on individuals’ baseline learning rate

To examine the causal relationship between catecholamine activity and learning rate, we used a within-subject placebo-controlled pharmacological manipulation of central NE and cortical DA activity. Several previous studies have shown that catecholaminergic drug effects are strongly baseline-dependent, such that they depend on an individual’s arousal state or baseline level of catecholamine activity [30,51–55]. This is consistent with the idea that the relationship between catecholaminergic activity and neurocognitive function is not monotonic but follows an inverted U-shape [25,56]. Therefore, we tested for atomoxetine effects at the group level, as well as for baseline-dependent atomoxetine effects.

**Fig 5:**
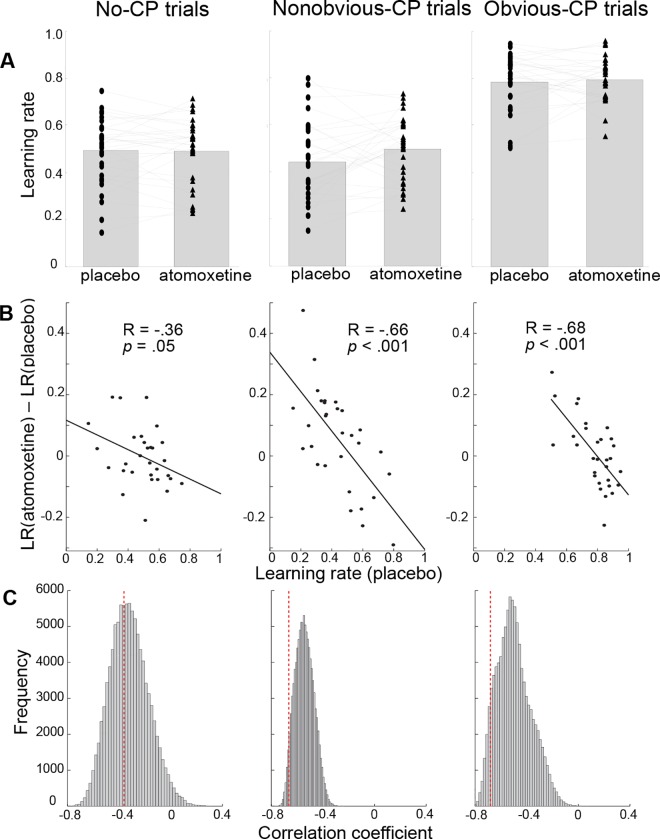
Baseline-dependent atomoxetine effects on learning rate. A. Each participant’s average learning rate in the placebo and atomoxetine session, separately for the trials with no change point, nonobvious change points, and obvious change points. The dots, connected with lines, represent individual participants’ learning rates in each session, and the bars indicate the group-mean learning rate. B. Across-subject relationship between learning rate in the placebo session and the atomoxetine effect on learning rate, separately for the trials with no change point, nonobvious change points, and obvious change points. C. Permutation distributions (100,000 permutations) of the correlation coefficients expected based on regression to the mean. Our observed correlations are indicated by red lines.

At the group level, none of the effects reported so far differed between the placebo and atomoxetine session: there were no main effects of treatment on learning rate (*p* = .89; Fig 5A) or P3 amplitude (*p* = .82; Fig 3A), and treatment did not interact with other task variables in any of the above-reported regression analyses (all *p*’s > .38). The hazard rate parameter, obtained by fitting the normative model to each participant’s predictions, did not differ between the placebo and atomoxetine sessions either (mean estimated hazard rate = .31 vs. .33, respectively, *t*(29) = .64, *p* = .5). Note that the model-estimated hazard rate was higher than the actual proportion of change points, which was .08 (see Methods), suggesting that participants overestimated the frequency at which change points occurred.

To test for potential baseline-dependent atomoxetine effects on learning rate, we next examined whether the atomoxetine effects on learning rate depended on participants’ learning rate in the placebo session (i.e., their ‘baseline’ condition). Specifically, we computed the across-subject correlation between the mean learning rate in the placebo session (LR_placebo_) and the change in mean learning rate in the atomoxetine compared to the placebo session (LR_atomoxetine_—LR_placebo_). Because we expected that atomoxetine effects on learning rate would be strongest following change points (during high unexpected uncertainty) and would also differ as a function of the obviousness of the change point, we computed mean learning rates separately for the trials on which no change point occurred, the trials on which an obvious change point occurred (change point outcome > 2 SDs from previous mean; 6.4% of all trials; mean learning rate = .78), and the trials on which a less obvious change point occurred (change point outcome < 2 SDs from previous mean; 2% of all trials; mean learning rate = .48). Note that learning rate was much lower following non-obvious than obvious change points, probably because it was more ambiguous whether or not a change point occurred on the non-obvious change-point trials. Also, the average normative learning rates on obvious and nonobvious change-point trials, as computed by our model (see Methods), were .87 and .26, respectively; hence participants used somewhat lower-than-optimal learning rates following obvious change points and higher-than-optimal learning rates following non-obvious change points. Importantly, there were negative across-subject correlations between the atomoxetine effect on learning rate and learning rate in the placebo session (Fig 5B). Thus, atomoxetine increased learning rates in participants with low baseline learning rates, but decreased learning rates in participants with high baseline learning rates. Importantly, this negative correlation was stronger on (obvious and non-obvious) change-point trials than on trials on which no change point occurred. There was no significant baseline-dependent atomoxetine effect on the hazard rate parameter (correlation = -0.23, *p* = .22).

Regression to the mean also predicts a negative correlation between learning rate in the placebo session and the atomoxetine effect on learning rate. To test whether there were baseline-dependent atomoxetine effects above and beyond those predicted by regression to the mean, we performed two additional analyses. First, we performed permutation analyses to obtain the distribution of correlation coefficients predicted exclusively by regression to the mean. This was done by computing the above-described across-subject correlation 100,000 times, each time using randomly assigned ‘placebo’ vs. ‘atomoxetine’ labels for the 2 sessions of each participant. We then compared our observed correlation coefficient (reflecting the combined effects of regression to the mean and potential baseline-dependent atomoxetine effects) against this permutation distribution. This analysis suggested that the baseline-dependent atomoxetine effect on learning rate was stronger than expected based on regression to the mean for the change-point trials (proportion of permutations below observed correlation = 0.04 and 0.07 for the nonobvious and obvious change-point trials, respectively) but not for the trials on which no change point occurred (proportion of permutations below observed correlation = 0.42; Fig 5C). Second, a baseline-dependent atomoxetine effect that is larger than expected based on regression to the mean should produce higher across-subject variance in learning rate in the placebo session than in the atomoxetine session [57]. We tested this prediction using Pitman’s test of equality of variance in paired samples [58]. The across-subject variance in learning rate was indeed higher in the placebo than the atomoxetine session for the obvious change-point trials (0.015 vs. 0.009, *t* = 3.3, *p* = .002) as well as the nonobvious change-point trials (0.029 vs. 0.019, *t* = 2.6, *p* = .01), but did not differ between sessions for the trials on which no change point occurred (0.021 vs. 0.020; *t* = .37, *p* = .72). The results from these two analyses suggest that the baseline-dependent atomoxetine effect on learning rate following change points is unlikely merely due to regression to the mean. Instead, it suggests that atomoxetine affected belief updating following outcomes that signal a potential change point, and that the direction of this effect depended on participants’ baseline learning rate.

We also examined whether the baseline-dependent atomoxetine effect on learning rate could be explained by atomoxetine effects on subjective state (alertness, calmness and contentment, which were measured once during each session, see Methods). To this end, we used partial correlations to test the across-subject relationship between learning rate in the placebo session and the atomoxetine effect on learning rate, while controlling for the three subjective-state measures in the placebo session and for the atomoxetine effect on each subjective-state measure. Controlling for subjective state in fact led to a small increase in the strength of the negative correlations between the atomoxetine effect on learning rate and learning rate in the placebo session (*R* = -.56, *p* = .02; *R* = -.76, *p* < .001; and *R* = -.72, *p* = .001, for the no change-point, nonobvious change-point and obvious change-point trials, respectively). This suggests that the baseline-dependent atomoxetine effects cannot be explained by atomoxetine effects on subjective state.

Finally, we tested for baseline-dependent atomoxetine effects on P3 amplitude. There were negative across-subject correlations between P3 amplitude in the placebo session and the atomoxetine effect on P3 amplitude (*R* = -.46, *p* = .02; *R* = -.54, *p* = .003: and *R* = -.57, *p* = .002 for the no change-point, nonobvious change-point and obvious change-point trials, respectively). However, permutation analyses showed that these correlations did not differ from those predicted by regression to the mean (proportion of permutations below observed correlations were 0.52, 0.60 and 0.37 for the no change-point, nonobvious change-point and obvious change-point trials, respectively). Pitman’s test of equality of variance in paired samples corroborated the absence of a baseline-dependent atomoxetine effect on P3 amplitude, as it showed that the across-subject variance in P3 amplitude did not differ between the two sessions (*t*’s < .5 for the change-point, nonobvious change-point and obvious change-point trials). Note that individual differences in skull thickness and brain morphology—which are unrelated to catecholamine activity—have a strong influence on baseline P3 amplitude [59–61]. Therefore, these variables are likely to obscure potential baseline-dependent atomoxetine effects on P3 amplitude that are due to catecholamine effects. Importantly, skull thickness and brain morphology can conceal between- but not within-subject P3 effects, as is evident from the absence of significant across-subject correlations between learning rate and P3 amplitude in either session (*p*’s > .3), despite strong within-subject relationships between P3 amplitude and learning rate (Fig 3C).

## Discussion

The present research provides novel evidence for a role of the human catecholamine systems in learning rate regulation. First, trial-to-trial variation in P3 amplitude—an index of phasic stimulus-evoked catecholamine release in the cortex [35–37]—mediated the effect of prediction-error magnitude on learning rate, suggesting that the phasic catecholamine response following surprising outcomes drives subsequent increases in learning rate. Second, two latent variables that together determine learning rate according to a previously-established computational model [4]were reflected in P3 amplitude. Third, a pharmacological manipulation of NE and DA activity had baseline-dependent effects on learning rate following unsignaled task changes, but not during periods of stable task contingencies, suggesting that NE and DA have a causal role in the adjustment of learning rate following environmental change.

We used a normative model [4,5,21] to dissociate three latent variables that jointly determine trial-specific learning rate in the current task: (i) change-point probability, which reflects the unexpectedness of an observation and hence the likelihood of environmental change; (ii) relative uncertainty, which reflects the uncertainty about the current state of the environment and hence the informational value of new observations (this has also been referred to as estimation uncertainty [62]); and (iii) hazard rate, which reflects one’s prior beliefs about the frequency of environmental change. Change-point probability and relative uncertainty are updated on each trial, and have previously been related to pupil change and average pupil size, respectively, during the outcome-viewing period in this task [21]. Hazard rate is fixed across trials but varies across participants. Our atomoxetine manipulation did not affect hazard rate, suggesting that catecholamine activity does not regulate prior beliefs about environmental volatility. However, trial-to-trial variation in P3 amplitude was sensitive to both change-point probability and relative uncertainty, and mediated the effects of both variables on learning rate, suggesting that the phasic release of NE and/or DA regulates learning rate as a function of both the unexpectedness and the informational value of new outcomes.

The sensitivity of the P3 to change-point probability is broadly consistent with findings from previous model-based EEG studies that trial-to-trial fluctuations in centroparietal P3 amplitude can be explained by the degree of surprise associated with the eliciting stimulus [47], and by various probabilistic and sequential effects across different time scales [63]. In addition, the sensitivity of the P3 to relative uncertainty is compatible with previous accounts that the P3 is sensitive to the amount of information conveyed by a stimulus [64,65]. Finally, our finding that P3 amplitude is predictive of learning rate fits nicely with a prevalent account of the functional significance of the P3, the context-updating hypothesis, according to which the function of the P3 process is to update one’s expectations about the current task context [66,67].

Via which mechanisms could a phasic catecholamine response following surprising or highly informative outcomes cause a transient increase in learning rate? There is a wealth of neurophysiological evidence that catecholamines boost the efficacy of synaptic interactions between neurons [9,68], thus increasing the gain of processing in cortical circuits responsible for task performance [25,69]. By selectively increasing gain following unexpected outcomes, the catecholamine systems could promote belief updating in a strongly stimulus-driven manner. This catecolaminergic modulation of learning rate may be similar in nature to the mechanism via which phasic NE and DA activity are thought to modulate attention, perception, and other types of learning [11,25,31,70–72].

P3 amplitude was a partial rather than a full mediator of the effects of prediction error, change-point probability and relative uncertainty on learning rate, implying that the process indexed by P3 amplitude is a significant, but certainly not the only, mediator of these effects. Other likely mediators are top-down processes such as the interpretation of prediction errors—e.g., the attribution of prediction errors to change points or to random noise—related to one’s internal model of the environmental dynamics. Previous fMRI studies have implicated the dorsal anterior cingulate cortex (ACC) in the updating of internal models [6] and volatility-driven learning rate adaptation [3], suggesting a role of this region in a more top-down type of belief updating. Interestingly, it has been shown that LC input to the ACC in rats leads to a suppression of model-based strategies, and a transition to a more stochastic choice mode that is independent of the ACC [73]. The respective contributions of the LC-NE system and the ACC, and their interactions, in regulating learning rate is an important topic for further investigation. Also, we focused on an environment that was dynamic in the sense that the *mean* of the outcome-generating process changed at unsignaled moments. The role of neuromodulatory systems in learning-rate regulation as a function of meta-level dynamics, such as changes in volatility or within-block changes in noise level, remains to be investigated.

Our results do not speak to where in the brain the surprise and uncertainty values that drive learning rate are computed. It has been proposed that the LC receives uncertainty information via afferent projections from cortical areas, including the ACC [22], which is supported by the existence of anatomical projections from frontal brain regions to the LC [25,74,75]. Several recent fMRI studies have started to examine where in the brain various types of uncertainty information are encoded [3,5,62,76]. One of these studies, for example, provided evidence that unexpected uncertainty (which we refer to as change-point probability) is represented in a brain network including the posterior cingulate cortex and hippocampus, whereas estimation uncertainty (which we refer to as relative uncertainty) is represented in another network including the ACC [62]. In that same study, unexpected uncertainty was also associated with sustained deactivation of a brainstem region that may correspond to the LC. Given the inverse relationship between tonic and phasic LC activity, this may suggest that increased unexpected uncertainty is associated with lower tonic LC activation in combination with larger stimulus-evoked phasic LC responses [25]. However, because LC activation is challenging to identify using standard neuroimaging methods [77], the activation in the vicinity of the LC must be interpreted with caution [78].

The effects of our atomoxetine manipulation on learning rate following change points depended on participants’ baseline (i.e., placebo session) learning rate: atomoxetine increased learning rate in participants with low baseline learning rates but decreased learning rates in participants with high baseline learning rates. Similar baseline dependencies have been reported for receptor-specific noradrenergic drug effects [51,52] and for dopaminergic drug effects [79,80]. This baseline dependency may explain why previous studies did not find catecholaminergic drug effects on behavior at the group level [81,82]. Thus, taking into account inter-individual variation is crucial in pharmacological studies of the NE and DA systems.

Our conclusions regarding the role of catecholamine activity in learning-rate regulation rely on the assumption that the P3 is an index of phasic catecholamine release in the cortex. The link between the phasic LC-NE response and the centroparietal P3 is supported by a wealth of neurophysiological evidence [35–37,83,84]. This link may in part be mediated by DA release in the cortex, where noradrenergic terminals co-release DA [39]. Indeed, a few studies have reported evidence that DA agents affect the centroparietal P3 to unexpected and novel stimuli, although primarily when these were task-irrelevant [38,85,86]. This link between catecholamine release and the P3 is not exclusive, because the centroparietal P3 is also modulated by pharmacological manipulations of the cholinergic system [87,88]. However, it is as yet unclear whether this reflects a direct effect on cortical acetylcholine release or results from mutual interactions between the basal forebrain and LC.

Our P3 measure and the effects of our atomoxetine manipulation are nonspecific with regard to the roles of NE vs. DA, because (i) the NE transporter is responsible for the reuptake of both DA and NE in the cortex [40–42], (ii) locus coeruleus activity results in the co-release of DA from noradrenergic terminals [39], and (iii) there are bidirectional projections between the dopaminergic nucleus ventral tegmental area and the noradrenergic locus coeruleus [89]. Furthermore, both systems are sensitive to uncertainty and have similar, partially overlapping post-synaptic effects (e.g., an increase in neural gain [ref 65]). Our findings are broadly consistent with the recent proposal that DA balances bottom-up sensory information and top-down prior beliefs during active inference [29]. With regard to NE, our findings corroborate the notion that NE signals ‘unexpected uncertainty’ arising from unanticipated changes in environmental contingencies, but not ‘expected uncertainty’ arising from known unreliability in these contingencies [11] (in our case, related to the noise in the generative process). It has been shown that DA also codes for uncertainty [90,91], but little is known about the exact forms of uncertainty that DA codes for. Thus, follow-up studies, perhaps using direct recordings in animal models [92], will be needed to disentangle the specific contributions of NE and DA to the effects revealed in this study.

Another open question is to what extent the observed atomoxetine effects reflect modulations of phasic (stimulus-evoked) versus tonic (spontaneous) catecholamine activity. NE transporter blockers such as atomoxetine have two opposing effects on catecholamine activity: while the reuptake inhibition increases catecholamine levels in the forebrain, the indirect activation of inhibitory α2-autoreceptors reduces activity of the LC itself [45]. The net effect of these two actions likely depends on the atomoxetine dose and on an individual’s baseline catecholamine activity. A recent study in rats found that atomoxetine reduced baseline LC activity while preserving the stimulus-evoked phasic LC response, thereby producing an increase in the phasic-to-tonic ratio of LC activity [93] which may effectively enhance neural responses to stimuli that evoke large LC responses. A similar atomoxetine effect on LC activity in our study may explain the specific atomoxetine effect on learning rate following change-point outcomes. Furthermore, atomoxetine effects on the phasic-to-tonic ratio of LC activity may depend on someone’s natural pattern of LC firing, possibly according to an inverted-U shape function, such that atomoxetine increases this ratio (more phasic) in people who naturally have relatively small phasic LC responses but decreases this ratio (more tonic) in people who naturally have large phasic LC responses. Such an inverted-U effect may underlie the baseline-dependence of atomoxetine effects on learning rate.

In sum, our results provide novel evidence that catecholamine systems are involved in learning-rate regulation, and encourage future studies to delineate the relative contributions of NE and DA and examine the underlying mechanisms of action.

## Methods

### Participants

Thirty-two healthy participants (mean age = 22.9, range = 18–28; 21 females) took part in the study in return for €135, plus a variable performance-related bonus of 6 to 20 euros. Exclusion criteria included history or presence of psychiatric disease and evidence of relevant clinical abnormalities. All participants provided informed consent, and the study was approved by the medical ethics committee of the Vrije Universiteit Amsterdam. Two participants were excluded from all analyses because of their poor performance on the predictive-inference task, and three additional participants were excluded from the EEG analyses because of excessive artifacts in their EEG data (see below). Thus, our behavioral and EEG analyses were based on 30 and 27 participants, respectively.

### General procedure

All participants took part in two experimental sessions, separated by one week. The two sessions took place at the same time of the day. In one session they received a single oral dose of 40 mg of the selective NE transporter blocker atomoxetine, and in the other session they received placebo, according to a double-blind, randomized, crossover design. At t = 75 minutes after drug administration, roughly corresponding with peak plasma concentrations of atomoxetine [94], participants performed a 30-minutes visual novelty-oddball task (results will be reported elsewhere), followed by the predictive-inference task. We recorded participants’ EEG throughout the experimental tasks. For 25 of the participants, we also measured subjective state 75 minutes after drug administration during each session, by means of visual analogue scales measuring alertness, calmness and contentment [95]. Ratings of alertness, calmness or contentment did not differ between the placebo and atomoxetine session (*t*(48) = 1.0, *p* = .31, *t*(48) = 0.74, *p* = .46 and *t*(48) = 1.1, *p* = .26, respectively)

### Predictive-inference task

During this 30-minutes task, participants repeatedly predicted the next location on a horizontal number line that ranged from 0 to 300 in units of 1 [4,21]. The number-line location on each trial was determined by the following number-generating process. On each trial, a number was randomly drawn from a Gaussian distribution, the mean of which changed at unsignaled moments—referred to as change points. The probability of a change point was 0.10 on each trial, except for the first 3 trials after the previous change point on which this probability was 0. When a change point occurred, a new mean for the number-generating distribution was randomly drawn from a uniform distribution ranging from 0 to 300 in units of 1. The SD of the number-generating distribution was constant within each experimental block of 200 trials, but varied across blocks. In each experimental session, participants completed two 200-trial blocks. The SDs of the number-generating distributions in the two blocks were 10 (low noise) and 25 (high noise). We used 4 instantiations of this number-generating process—2 for each SD—hence all participants experienced exactly the same sequences of outcomes, in counterbalanced order (Fig 1A).

Throughout the task, a horizontal number line, ranging from 1 to 300, was presented on the screen (Fig 1B). At the start of each trial, participants predicted the next number by selecting a specific location on the number line, using a mouse, after which a small green oval was displayed underneath the selected location. One second later, an arrow accompanied by the actual number outcome on that trial was displayed in red above the corresponding location on the number line, and the difference between this outcome and the participant’s prediction was indicated by a gray bar. Half a second later, the next trial started and participants updated their prediction. To ensure that learning rates were always in the 0–1 range, we constrained participants’ prediction space to the interval in between (and including) their previous prediction and the most recent outcome (cf. [4,21]; data collected using this constraint does not differ substantially from tasks where the constraint is not applied [5]).

To make the task more engaging, we embedded the task in a cover story in which the number line represented the earth, and the number outcomes reflected the locations of missile attacks from outer space directed at particular locations on earth. To this end, a picture of a planet above a layer of asteroids was displayed above the number line. We instructed participants that on each trial they could place a “laser shield” (the green oval) above a specific location on earth (their prediction) to prevent that location from being hit. In order to make the number-generating process as transparent as possible—i.e., to minimize structural uncertainty—we gave participants the following two additional instructions: (i) on their way to earth the missiles pass through an asteroid layer, causing random deflections of their direction and therefore trial-by-trial variation in their impact locations; and (ii) the location on earth at which the missiles are aimed changes at unpredictable moments. These instructions provide intuitive information about the SD (noise) of the number outcomes and the occasional change points, respectively. Before starting the experimental blocks, participants completed two practice blocks of 30 trials each.

We defined the prediction error on each trial as the difference between the observed and predicted number outcome, i.e., prediction error(t) = outcome(t)–prediction(t), and the learning rate as the participant’s prediction update as a fraction of the most recent prediction error, i.e., learning rate(t+1) = [Prediction(t+1)–Prediction(t)] / prediction error(t). Two participants fully updated their predictions to the most recent outcome on nearly all trials in both sessions, suggesting a misunderstanding of the task structure. As is usual for such cases (e.g.[5]), we excluded these participants from further analyses.

### Normative model

We also used an approximately Bayesian learning model that has been shown to capture key aspects of participants’ performance on the predictive-inference task [4,21]. The model updates beliefs about the current outcome-generating distribution according to a delta rule with a dynamic learning rate:

$$B_{t+1}=B_{t}+\alpha_{t}\times \delta_{t}$$

where B_t_ is the model’s prediction about the mean of the generative distribution, α_t_ is the learning rate, and δ_t_ is the prediction error (i.e., difference between observed and predicted outcome) on trial t. In models with a static learning rate, the current belief is a weighted average of previous outcomes, with the weights of previous outcomes decaying exponentially into the past. In contrast, in our model α_t_ is determined on each trial by two variables, change–point probability (Ω) and relative uncertainty (τ), according to:

$$\alpha_{t}=\Omega_{t}+{(1-\Omega }_{t})\tau_{t}$$

Ω_t_—which has been referred to as unexpected uncertainty in previous studies [11,62]—reflects the posterior probability that a change point has occurred since the previous trial, which increases transiently following surprising outcomes. The model computes Ω_t_ following each new outcome (X_t_) as a function of the likelihood of that outcome if a change point had occurred and the likelihood of that outcome if a change point had not occurred:

$$\Omega_{t}={\frac{U(X_{t}|0,300)H}{U(X_{t}|0,300)H+N(X_{t}|B_{t},\sigma_{t}^{2})(1-H)}}_{}$$

where U is the uniform distribution from which X_t_ is generated if a change point occurred; N is the predictive normal distribution if a change point did not occur; B_t_ is the model’s prediction on trial t; σ^2^ is the total variance of the predictive distribution; and H is the hazard rate. The hazard rate is the proportion of trials on which a change point occurred (i.e., the prior probability of change points) which was 0.08 in our task.

The total uncertainty about the next outcome (σ^2^, i.e., the total variance of the predictive distribution) is the sum of the variance of the generative distribution (N^2^, i.e., noise) and the uncertainty about the mean of the generative distribution. While outcome uncertainty due to noise is constant within each task block, uncertainty attributable to imprecise knowledge of the mean of the generative distribution decreases with each outcome observation in a stable regime. Just as the gain in a Kalman filter, appropriate learning in our task depends on the proportion of total outcome uncertainty that is due to an imprecise estimate of the generative mean, and we define relative uncertainty (τ) as this proportion. On each trial, relative uncertainty is computed according to the variance on the predictive distribution over generative means (a weighted mixture of change point and non-change point conditional distributions) according to the following equation:

$$\tau_{t+1}={\frac{\Omega_{t}\sigma_{N}^{2}+(1-\Omega_{t})\tau_{t}\sigma_{N}^{2}+\Omega_{t}(1-\Omega_{t})(\delta_{t}(1-\tau_{t}){)}^{2}}{\Omega_{t}\sigma_{N}^{2}+(1-\Omega_{t})\tau_{t}\sigma_{N}^{2}+\Omega_{t}(1-\Omega_{t})(\delta_{t}(1-\tau_{t}){)}^{2}+\sigma_{N}^{2}}}_{}$$

where the numerator reflects the variance on the predictive distribution over possible generative means and the denominator is the total outcome variance, which also includes the noise variance [5,21]. Relative uncertainty is computed in anticipation of each upcoming outcome and therefore reflects outcome-independent adjustments in learning [4].

We obtained per-trial estimates of change-point probability and relative uncertainty, by applying the model to each participant’s observed sequence of outcomes while fixing hazard rate (H) to the actual proportion of change-point trials (.08). Hazard rate can also be treated as a free parameter that is estimated by fitting the model to each participant’s prediction data, thereby capturing inter-individual variability in learning rate due to different prior expectations about the frequency of change points. To examine potential effects of atomoxetine on the hazard rate parameter, we fitted the model to each participant’s predictions in each session by minimizing the total squared difference between the participant’s and the model’s predictions, using a constrained search algorithm (fmincon in MATLAB).

### EEG recording and analyses

We recorded EEG from 128 scalp electrodes, placed according to the radial ABC system of BioSemi, and from the left and right mastoids. We measured the horizontal and vertical electro-oculogram (EOG) using bipolar recordings from electrodes placed approximately 1 cm lateral of the outer canthi of the two eyes and from electrodes placed approximately 1 cm above and below the participant's right eye. The EEG signal was pre-amplified at the electrode to improve the signal-to-noise ratio and amplified with a gain of 16x by a BioSemi ActiveTwo system (BioSemi, Amsterdam). The data were digitized with a sampling rate of 512 Hz

EEG data were processed using a combination of BrainVision Analyzer 2 (Brain Products) and Matlab (Mathworks), the latter via custom scripting and subroutines from the EEGLAB toolbox [96]. Continuous data were first re-referenced to the average of the left and right mastoid channels, and high-pass filtered to 0.1 Hz (12 dB/octave). Ocular artifacts were removed using a regression-based algorithm [97], after which the data were low-pass filtered up to 30 Hz (12 dB/octave). Noisy channels were then identified by visual inspection of signal variance and interpolated via spherical spline interpolation. Data epochs were extracted from 250 ms before to 1000 ms after outcome onset on each trial and baseline-corrected to the 250-ms interval preceding outcome onset. All epochs were then inspected for violations of amplitude (any sample from any scalp channel with an absolute voltage > 150 μV) and gradient (any scalp channel where absolute slope of a fitted line to the data was > 65 μV/s) artifact criteria. In cases where no more than 2 channels were identified as artifactual, those channels were interpolated and the associated epoch was retained for subsequent analysis; otherwise, that epoch was discarded. For three participants, more than 50% of epochs in one session were identified as artifactual (1 placebo, 2 atomoxetine) and these participants were excluded from all EEG analyses. A mean of 2.2 ± 2.3% of epochs for the placebo sessions and 6.3 ± 7.9% of epochs for the atomoxetine sessions were rejected for the remaining 27 participants. For all analyses, our measurement of the outcome-locked P3 component was based on the mean signal across a cluster of four centroparietal electrodes that was centered on the region of maximum component amplitude in the grand-average topography (corresponding to the location of CPz and Pz according the standard 10/20 measurement system). For single-trial analysis of the P3, waveforms were low-pass filtered to 6 Hz to enhance signal-to-noise and P3 amplitude was measured as the mean voltage between 340 and 520 ms post-outcome.

### Statistical analyses

We conducted multi-level regression and mediation analyses on single-trial measures of prediction error, learning rate and P3 amplitudes, using the Multilevel Mediation toolbox (http://wagerlab.colorado.edu/tools [48,98,99]). These analyses take into account trial-to-trial variation (within-subject effects; first level) and between-subject variation (second level) in the same model. To permit the use of the [0,1]-bounded learning rate as a dependent variable in these analyses, we performed a logit transformation on the learning rate values: learning rate_logit_ = ln(1/(1-learning rate)).

*Regression analyses*. We examined the linear effects of absolute prediction error, SD of the generative distribution, treatment (atomoxetine vs. placebo), and their interactions on learning rate_logit_ and P3 amplitude, using multilevel-regression analyses. Trials with prediction errors of 0 were excluded from the analysis on learning rate (2.0% of all trials), as participants could not update their prediction on those trials (see task description above). In the analysis on P3 amplitude, we included a binary regressor that indicated whether or not the prediction error was exactly zero.

In a separate regression model, we tested the linear effects of estimated change-point probability and relative uncertainty on P3 amplitude, while also including regressors for treatment, SD of the generative distribution, a binary variable indicating whether or not the prediction error was exactly 0, and the treatment x change-point probability and treatment x relative uncertainty interactions. Treatment order was included as a second-level regressor in all regression models.

*Mediation analyses*. We further examined the relationships between trial-to-trial variation in absolute prediction error, P3 amplitude and learning rate_logit_ using multilevel mediation. Mediation analyses test whether the relationship between an independent variable (X) and a dependent variable (Y) can be explained by a third variable (M; the mediator). Thus, rather than assuming a direct effect of X on Y, mediation analyses test whether X influences M, which in turn influences Y. A mediation model can be formally captured by a set of three regression equations:

1. *Y* = *cX* + *e*_*Y*_
2. *M* = *aX* + *e*_*M*_
3. *Y* = *bM* + *c’X* + *e’*_*Y*_

Here, Y, X, and M are data vectors containing trial-specific measures of the dependent, independent and (potential) mediator variables, respectively. *c* is the slope of the X-Y relationship (i.e., the estimated linear change in Y per unit change in X), *a* is the slope of the X-M relationship, *b* is the slope of the M-Y relationship controlling for X. *c’* is the slope of the X-Y relationship when controlling for M, which is referred to as the direct or non-mediated effect of X on Y. Finally, e_Y_ and e_M_ denote residual errors for Y and M controlling for X, and e’_Y_ denotes residual errors for Y controlling for X and M. Variable M is considered to be a significant mediator if its inclusion in the model significantly affects the slope of the X-Y relationship; that is, if the difference (*c*−*c*′)—which is equivalent to the product of coefficients *a* and *b*—is statistically significant.

We conducted three different mediation analyses. In our first mediation model, we used prediction error as the X variable, learning rate as the Y variable, and P3 amplitude as the M variable. Thus, this model tested whether (i) there was an effect of prediction error on P3 amplitude (path *a*); (ii) P3 amplitude was predictive of learning rate, when controlling for prediction error (path *b*); and (iii) the relationship between prediction error and learning rate was formally mediated by P3 amplitude, i.e. whether the relationship between prediction error and learning rate (path *c*) decreased when controlling for P3 amplitude (*c*-*c*’, equivalent to a*b). In two additional mediation models we replaced prediction error by the computational variables change-point probability and relative uncertainty (derived from the normative model) as the independent variable (in separate analyses).

Trials with prediction errors of 0 were excluded from all mediation analyses, and we tested for linear effects. We included treatment and the SD of the generative distribution as covariates, and treatment order as a second-level moderator, in all mediation models. We tested the significance of all effects using a bootstrap procedure (100,000 bootstrap samples).
